# Hemifacial Spasms or Focal Aware Seizures: The Role of Video-EEG

**DOI:** 10.7759/cureus.80099

**Published:** 2025-03-05

**Authors:** Juan A Moncayo, Walter R Duarte-Celada, Pamela Davila-Siliezar, Jannatul Ferdous

**Affiliations:** 1 Department of Neurology, Texas Tech University Health Sciences Center, Lubbock, USA

**Keywords:** all neurology, focal seizures, hemifacial spasms, video eeg, video-eeg monitoring

## Abstract

Hemifacial spasms (HFS) are common in neurological practice but can be mistaken for focal aware seizures without appropriate diagnostic workup. We present the case of a man in his 60s admitted for congestive heart failure (CHF) exacerbation, who developed left-sided weakness and HFS during hospitalization. Although his motor symptoms improved after receiving tenecteplase (TNK), the HFS persisted, prompting the use of continuous video-EEG to differentiate between cortical myoclonus and HFS.

Video-EEG is essential for distinguishing HFS from focal aware seizures, identifying high-frequency ipsilateral muscle artifacts in HFS versus interictal or ictal EEG patterns contralateral to facial contractions in seizures. This case highlights key features to aid neurologists in differentiating these conditions.

## Introduction

Hemifacial spasms (HFS) are a movement disorder characterized by persistent or brief twitching of the muscles innervated by the seventh cranial nerve, most commonly due to aberrant blood vessels (primary HFS) [1]. Although benign, this condition is frequently misdiagnosed [2] and can be difficult to differentiate from focal seizures. Video-EEG is a valuable tool for diagnosis and tailoring treatment.

An involuntary and paroxysmal twitching of facial muscles on one side of the face, due to abnormal firing of the ipsilateral facial nerve, characterizes HFS. However, cases of bilateral involvement have been reported [3]. The recommended treatment for HFS is botulinum toxin injections. Yoshimura DM et al., in their randomized controlled clinical trial, have shown what is widely accepted by neurologists worldwide: safety and a low profile for side effects, with objective improvement in 84 percent of patients and expected facial weakness in 97 percent [4].

Structural brain abnormalities are the most common causes of focal seizures. Nevertheless, they cannot always be identified. Hippocampal sclerosis is one of the most commonly associated causes of this disorder, mostly involving the hilar region, dentate gyrus, CA1, CA3, and CA4. Other structural causes that can be identified through neuroimaging include central nervous infections, cortical dysplasia, perinatal injury, head trauma, vascular malformations, autoimmune encephalitis (e.g., LGI1 encephalitis, which manifests with facio-brachial dystonic seizures), and stroke [5,6].

Some focal epileptic disorders can be confused with HFS. Two examples of such disorders are: 1) autosomal dominant nocturnal frontal lobe epilepsy, which occurs only during the non-rapid eye movement sleep stage. Also known as sleep-related hypermotor epilepsy, it has an onset in childhood or adolescence with preserved cognition and neurologic functions, and the EEG is often normal during interictal periods [7]. 2) Self-limited epilepsy with centrotemporal spikes, which often presents with facial and oral twitches along with sensory alteration [8].

## Case presentation

A man in his 60s with a history of prior stroke with residual dysarthria and coronary artery disease was hospitalized for exacerbation of chronic heart failure. Neurology was consulted due to worsening of his baseline left-sided weakness and the new onset of left HFS. On initial assessment, the National Institutes of Health Stroke Scale (NIHSS) score was 11, attributed to his inability to state his age and the current month, left facial droop, left-sided hemiparesis, dysarthria, and intermittent left HFS. A CT scan of the head ruled out intracranial hemorrhage, and the patient subsequently received tenecteplase (TNK).

The following day, MRI of the brain revealed no new ischemic insults but confirmed encephalomalacia in the right pons and an old right periventricular/basal ganglia stroke. A routine EEG conducted two hours later failed to capture the left HFS. Given the history of previous strokes, the possibility of focal epilepsy as a cause of postictal weakness, and the new onset of HFS, there was debate regarding the underlying etiology. Consequently, continuous video-EEG monitoring was initiated to capture the episodes. This revealed focal muscle artifact in the left frontal region without evidence of ictal, interictal, or slowing patterns (Video 1).

**Video 1:** EEG of hemifacial spasms: high-frequency discharges, consistent with muscle artifact, noted in the F7 and F3 leads, ipsilateral to the contraction of the orbicularis oculi, upper lip, and zygomaticus muscles (all muscles innervated by the facial nerve). Adapted from Moncayo JA et al.

The patient was diagnosed with HFS and recrudescence of prior stroke symptoms versus aborted stroke. Notably, CT angiography of the head and neck was unremarkable for vascular malformations.

Follow-up care was offered to the patient through our continuity clinic for the management of HFS if they were determined to be severe enough to impact his functioning and well-being. Unfortunately, the patient did not return for his follow-up appointment.

## Discussion

Focal seizures are commonly classified into neocortical and mesiotemporal types, based on electrographic findings and clinical semiology.

The International League Against Epilepsy (ILAE) categorizes focal seizures into several types: focal motor seizures, focal non-motor seizures, focal aware seizures, focal impaired awareness seizures, and focal to bilateral tonic-clonic seizures [9].

Neocortical seizures originate outside the temporal lobe and involve areas responsible for "higher" cortical functions. These regions are densely interconnected and, as Wagner et al. recently demonstrated, exhibit expanded circuits with the cerebellum, highlighting a codependence in processing. This relationship may explain subcortical and cerebellar depolarization and the associated semiology [10]. Neocortical seizures can occur with or without altered consciousness and may propagate to evolve into bilateral convulsive seizures. Their symptoms vary depending on the cortical region affected.

HFS in the presence of risk factors for focal aware seizures require careful differentiation, with video-EEG being the most effective diagnostic tool. Unlike routine EEG, video-EEG enables simultaneous analysis of electrographic data and clinical observations, enhancing diagnostic accuracy.

The distinguishing electrographic features of HFS and focal aware seizures are outlined in Table 1. Video-EEG identifies HFS by detecting high-frequency ipsilateral muscle artifacts corresponding to facial contractions, characterized by a lack of slowing, spreading, or rhythmic changes, and abrupt termination. In contrast, focal motor seizures typically show spreading to adjacent leads, postictal slowing, interictal or ictal patterns contralateral to facial contraction, and a gradual, rhythmic onset followed by slowing.

**Table 1:** Electrographic characteristics of hemifacial spasms (HFS) versus focal aware seizures. Adapted from Moncayo JA et al.

EEG Characteristics	HFS	Focal aware seizure
Lateralization	Ipsilateral to facial movements	Contralateral to facial movements
Pattern	Extremely high frequency discharges	Ictal or interictal, including slowing of EEG.
EEG slowing	Absent	Could be present, pre- or post-event, or even on the contralateral side
Spreading	Absent	Could be present
EEG changes onset and termination	Sudden onset and sudden termination	Rhythmic and gradual increase followed by slowing prior to ending abruptly

## Conclusions

This review highlights the clinical and diagnostic complexity of HFS and their differentiation from focal seizures, particularly focal aware seizures. The presented case of a man in his 60s underscores the diagnostic challenges in patients with overlapping neurological conditions, such as prior strokes and new-onset HFS. Video-EEG emerges as an indispensable diagnostic tool, offering superior precision over routine EEG by enabling the correlation of electrographic and clinical findings.

The clinical case emphasizes the importance of distinguishing HFS from focal aware seizures, as the underlying etiology directly guides treatment strategies, ranging from botulinum toxin for HFS to antiepileptic drugs for focal seizures.

Ultimately, the integration of neuroimaging, clinical history, and advanced EEG techniques is essential for accurate diagnosis and optimal patient outcomes, particularly in cases where differentiation between peripheral and central causes is crucial. This case serves as a valuable guide for neurologists in managing similar diagnostic dilemmas.
